# The risk of heat stress to South African emergency medicine registrars rotating through hospitals in warmer climate zones during summer months

**DOI:** 10.1007/s00484-025-02953-0

**Published:** 2025-06-18

**Authors:** MP Fitchett, JM Fitchett

**Affiliations:** 1 Division of Emergency Medicine, Faculty of Health Sciences, University of the Witwatersrand, Johannesburg, South Africa; 2 School of Geography, Archaeology and Environmental Studies, University of the Witwatersrand, Johannesburg, South Africa

**Keywords:** Humidex, Heat stress, Acclimatization, Registrar medical training, South Africa

## Abstract

Most South African Medical registrar programmes involve rotation between a number of teaching hospitals within a geographic region. For some of these programmes this includes a short rotation to a more remote hospital, in some instances located in a different province of the country, and distinct climate zone. This is the case for the University of the Witwatersrand Emergency Medicine programme, based in Johannesburg, which includes a two month remote rotation through Klerksdorp, a city located in the North West Province, with a markedly warmer climate, particularly during the summer months. The difference in climatic conditions, short duration of the rotation, and the lack of a defined period for acclimatisation heightens the risk of heat stress to these rotating Emergency Medicine registrars, particularly in residential environments. This holds a range of secondary risks, relating to the level of focus, agitation, and impact on sleep between shifts. In this study we use a collection of 3229 temperature and humidity readings taken over the period of a month in early summer in Klerksdorp. Using Humidex, a thermal comfort index for occupational health and safety, we quantify the risks of heat stress to acclimatised and unacclimatised workers, and the times of day of greatest heat risk. These findings can provide valuable direction in the scheduling of rest periods, the allocation of longer and shorter shifts through the day, and the provision of infrastructural adaptation to heat stress such as water coolers and air conditioning.

## Introduction

Exposure to high air temperatures for prolonged periods of time, or extremely high temperatures over short periods of time, reduces the physiological capacity of the human body to compensate for heat, posing a risk of heat stress (Lee 2014; Founda et al. 2019; Hosokawa et al. 2021). At extremes, heat stress can result in heat stroke and cardiovascular disease, with the potential for mortality (Lee 2014; Ngwenya et al. 2018). Controlled environment simulation studies have demonstrated that symptoms of more mild heat stress can include a reduction in quick and accurate decision making (De La Cruz et al. 2021), time perception and response times (Kingma et al. 2021), and risk-taking (Syndicus et al. 2018). Globally, heightened nighttime temperatures are associated with sleep loss (Minor et al. 2022). Statistical analyses in South Africa have further demonstrated that heat stress heightens the incidence of assault (Schutte et al. 2021) and other violent crimes (Potgieter et al. 2022). This poses a cumulative risk to doctors, where their own performance is compromised by heat, while patient loads may increase from both trauma and medical cases exacerbated by hot weather. Despite medical doctors likely being more aware of the risks of heat stress than the general public, there is little research and guidance on how to manage these risks for medical personnel in temporary homes when a basic duty of care necessitates work, and thus rest, across all hours.

**Fig. 1:**
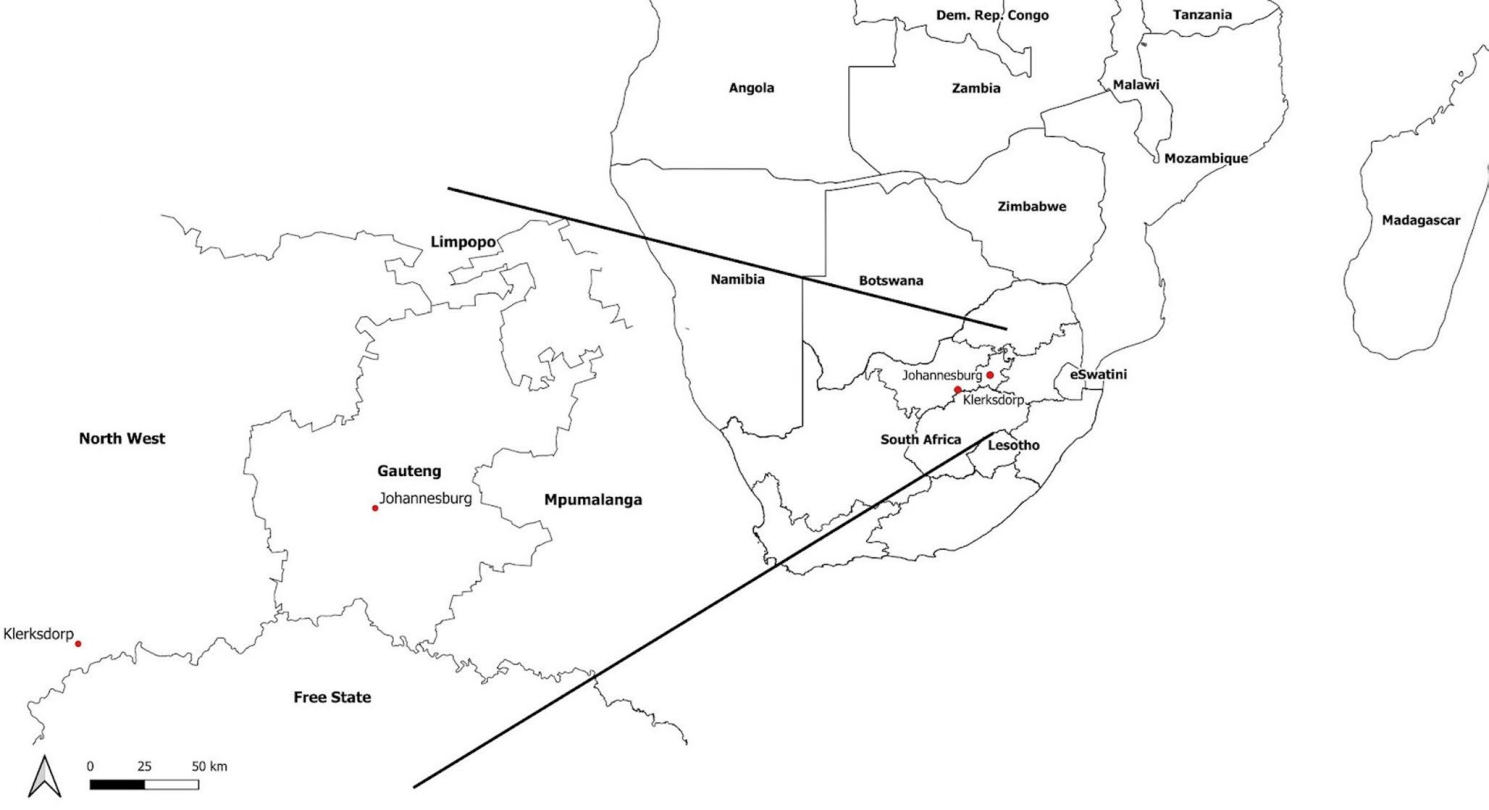
Study site map indicating the locations of Johannesburg and Klerksdorp, South Africa

In South Africa, registrar programmes for the specialisation of medical doctors, following their initial undergraduate medical degrees, involve rotation through a range of teaching hospitals and clinics that are associated with the university where they are registered for their training (Cooke et al. 2011). Gauteng is the smallest, yet most populous, province in the country. The majority of rotations for the University of the Witwatersrand registrar programmes take place within the southern half of the Gauteng Province. At present, the one exception is a two-month rotation at Klerksdorp-Tshepong hospital complex, located 170 km west-north-west of Johannesburg in North-West Province (Fig. 1). Klerksdorp is classified as a BSh climate (Semi-arid, hot), whereas Johannesburg is classified as a BSk (Semi-arid, cold), with an average temperature difference of 3–4 °C in daytime temperatures. During summer months, temperatures in Klerksdorp frequently exceed 25 °C, and statistically significant increases in heat extremes computed by the Expert Team on Climate Change Detection Indices (ETCCDI) have been calculated for both summer and winter months (van der Walt and Fitchett 2021). The risks of heat stress are heightened among unacclimatized persons, and under conditions of physical exertion (Périard et al. 2021). The CDC recommended period for acclimatisation is 1–2 weeks (NIOSH 2017).

The absence of a defined period for acclimatisation when moving to Klerksdorp, the short duration of the registrar rotations, and the physical exertion required in a hospital setting, warrant the investigation of the level of heat stress risk to these unacclimatized workers. In this study we explore 3229 records of Humidex occupational health and safety thermal comfort index scores computed at 15-minute intervals over a one-month period in early summer in an indoor, shaded residential setting in Klerksdorp to quantify the risk of heat stress to rotating registrars in their time off duty. On the basis of these results, we make recommendations pertaining to adaptation both in terms of shift scheduling and infrastructural improvements.

## Methods

Air temperature, dew point temperature and relative humidity were recorded automatically at 15-minute intervals using a Lascar EL-SIE-2 + high accuracy data logger, from GMT + 2 (local time zone) 16:00 on 26/11/2023 until 08:45 on 30/12/2023. Data were recorded at GMT time, and local time zones converted. The data collection period spans the early summer season in the North-West Province of South Africa (van der Walt and Fitchett 2020). The logger was mounted in a PVC stand to prevent conductive heat transfer, and situated in the middle of an indoor residential space where a rotating doctor was residing (and which is a popular location for many rotating doctors, with the property housing multiple doctors at any time), located at 26.851582 S, 26.666973E, in full shade. No air conditioning was available in this space, and airflow was facilitated by means of a small fan.

These data were used, in the first instance, to calculate Humidex, a thermal comfort index developed by the Canadian Centre for Occupational Health and Safety (CCOHS 2024). Importantly, Humidex provides an output classification that is calibrated by the level of acclimatisation and exertion undertaken (Table 1), providing a more nuanced interpretation of the thermal comfort to stress continuum than those using the Wet Bulb Globe Temperature (Rana et al. 2013; Teyton et al. 2022).

**Table 1:**
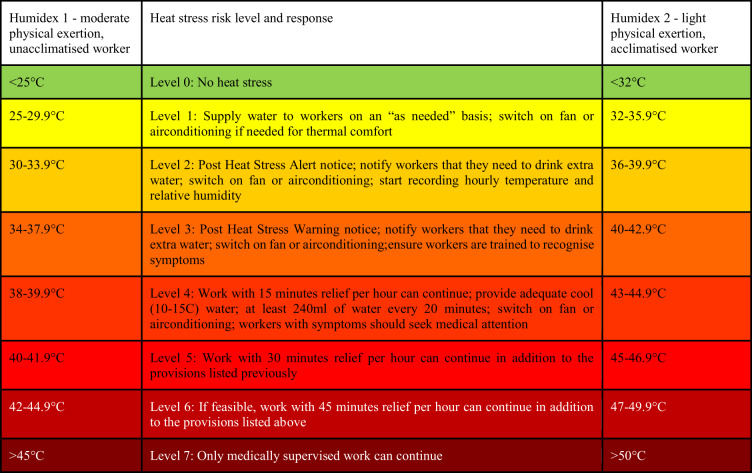
Humidex classifications for acclimatised and unacclimatised persons

Source: Occupational Health Clinics for Ontario Workers - “Humidex Based Heat Response Plan

Humidex is calculated by:

$$\:Humidex=T+\:\frac{5}{9}\:\times\:\:\left(6.112\times\:{10}^{7.5\times\:\frac{T}{237.7+T}}\times\:\frac{H}{100}-10\right)$$

Where

T: air temperature (°C)
H: relative humidity (%)

The output variables are classified on the basis of the level of exertion and acclimatisation as Humidex 1 and 2, on the basis of which occupational recommendations are made:

For each 15-minute interval, the Humidex score was calculated, and the classification assigned for both Humidex 1 and Humidex 2. The proportional representation of each of the thermal stress severity levels was then computed for the two output classifications to quantify the level of heat stress that acclimatised and unacclimatized persons are exposed to in the early summer. Thereafter, the data were classified by the time of day, whereby “morning” refers to 06:00–10:59, “midday” refers to 11:00–13:59, “afternoon” refers to 14:00–17:59, and “night” refers to 18:00–05:59, all in local GMT + 2 time, to explore the incidence of each level of heat stress through the day, and the time of greatest heat risk.

In the interests of comparison, the Heat Index, developed by the United States of American National Weather Service, was also computed and the output scores classified. Similar to humidex, the Heat Index integrates measurements of air temperature and relative humidity to quantify the physiological experience of heat, and classify the risk of thermal stress (Anderson et al. 2013). The Heat Index is calculated as outlined by Robinson (2001), by:

$$\begin{array}{c}\begin{array}{c}H_i=16.023+0.185212T+5.37941R-0.100254TR+\left(9.4169\times\:10^{-3}T^2\right)\\+\left(7.28898\times\:10^{-3}R^2\right)+\left(3.45372\times\:10^{-4}T^2R\right)-\left(8.14971\times\:10^{-4}TR^2\right)\\+\left(1.01202\times\:10^{-5}T^2R^2\right)-\left(3.8646\times\:10^{-5}T^3\right)+\left(2.91583\times\:10^{-5}R^3\right)\end{array}\\+\left(1.42721\times\:10^6T^3R\right)+\left(1.97483\times\:10^{-7}{TR}^3\right)-\left(2.18429\times\:10^{-8}T^3R^2\right)\\+\left(8.43296\times\:10^{-10}T^2R^3\right)-\left(4.81975\times\:10^{-11}T^3R^3\right)+0.5\end{array}$$

Where

T: temperature (in °F)
R: relative humidity (in %)

As air temperature data are captured in °C, these are converted to °F. Output values are in °F, but additionally further re-converted to °C and classified (Table 2) for comparison to the Humidex outputs.

**Table 2:**
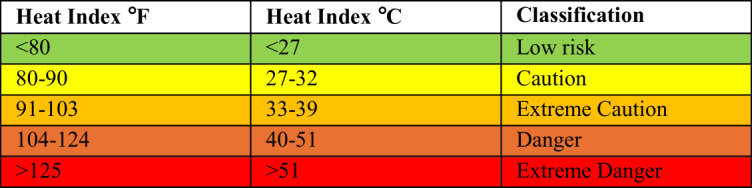
Heat Index Score Classifications

Source: USA National Weather Service

## Results

During the period of data collection, a mean air temperature of 28.96 °C was recorded (SD = 2.49), spanning a maximum of 33.92 °C on 27/11/2023 at 18:00 and 18:15, to a minimum of 22.05 °C on 12/12/2023 at 02:15. During this same period, mean temperatures in Johannesburg, measured from the South African Weather Service station at OR Tambo International Airport, were a considerably lower 21.0 °C. Temperatures in Johannesburg in the month prior to the rotation averaged 18.7 °C. These differences in conditions between Johannesburg and Klerksdorp are consistent with their climate zones, as described above.

There was a comparatively high average relative humidity for a semi-arid region, of 47.24% (SD = 7.63), reflective of the summer rainfall conditions (Roffe et al. 2019). Due to the convective nature of these storms, often associated with the Tropical Temperate Trough (Tozuka et al. 2014), there was a range from a maximum of 63.2% on 27/12/2023 from 22:45 − 23:30 to a minimum of 31.7% on 3/12/2023 from 23:15–23:30. These yield a mean Humidex score for the period of data collection of 33.82 °C (SD = 3.45), classified as level 1 heat stress for acclimatised workers, and level 2 heat stress for unacclimatised workers. Humidex values range from a maximum of 42.26 °C on 21/12/2023 at 17:00, classified as a level 3 heat stress for acclimatised workers and a level 6 heat stress for unacclimatised workers, to a minimum of 24.50 °C on 12/12/2023 at 06:00, which is classified as no heat stress risk for both acclimatised and unacclimatised workers.

**Fig. 2:**
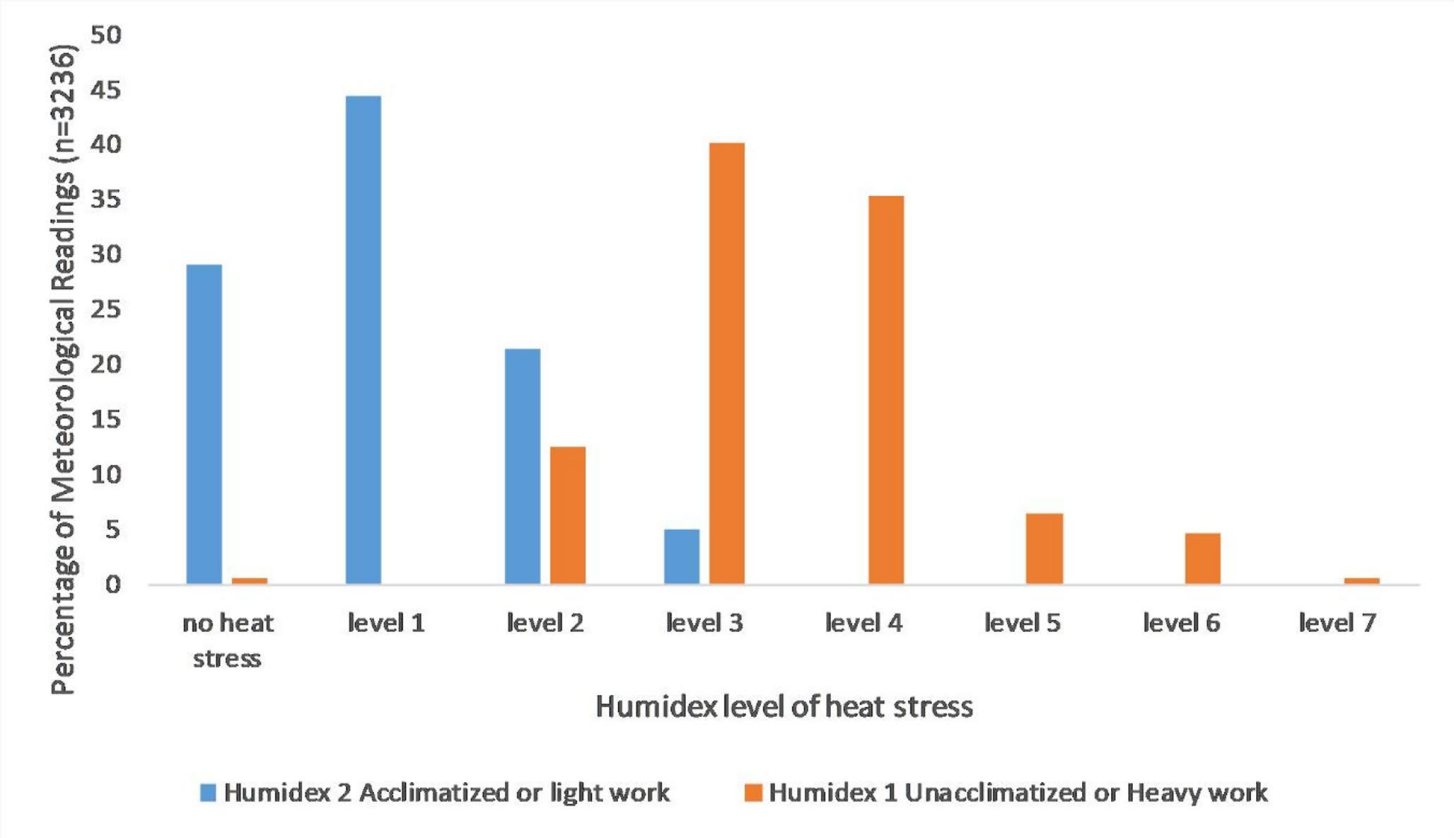
Percentage of humidex scores classified under each heat stress level

Across the 3229 Humidex scores calculated over a period of more than a month, the differences in the classification ranges for acclimatised and unacclimatised persons become apparent (Fig. 2). For an acclimatised worker “no heat stress” is experienced for 29.1% of the readings. Level 1 heat stress, for which the guidance is to supply persons with, or obtain individually, water on an as-needed basis, dominates the record at 44.4% of readings. The heat stress for acclimatised or persons decreases in incidence thereafter, with 21.4% of readings at a level 2 heat stress risk, and the remaining 5.1% at a level 3 heat stress risk. Accordingly, the maximum level of intervention needed would be to post a heat stress notice, notify persons that they need to drink extra water, and ensure that persons are trained to notice symptoms. While medical doctors in specialist training programmes should be able to notice the symptoms of heat stress in themselves and their colleagues living on the property, the former two interventions are not commonly implemented.

For registrars rotating through Klerksdorp in the summer months, the category of ‘unacclimatised’ would be more representative, particularly in the first two weeks of the rotation. Humidex 2 is therefore a more representative classification framework for the first two weeks of the rotation, following which Humidex 1 would be appropriate for persons who are largely sedentary as acclimatisation has taken place. Humidex 2 would remain appropriate for conditions of moderate to heavy physical exertion, which still could apply outside of the work setting in sporting, housework and transportation by cycling. For Humidex 2, only 0.5% of the readings for the period of data collection would be classified as “no heat risk”. The largest proportion of readings are classified as level 3 (40.1%) and level 4 (35.4%) heat risk (Fig. 2), the latter of which is suggested to be mitigated by any work, which could include housework or exercise, interspersed with 15 min relief per hour, adequate provision or intake of cool (10–15 °C) water, the consumption of at least 240 ml of water every 20 min, and persons with symptoms seeking medical attention. The Humidex classifications for this category extend to a level 7 heat stress (0.5% of the readings), for which only medically supervised activities should continue. While it could be argued that all work in a hospital environment is under medically supervised conditions, it is not unreasonable to imagine that the doctors’ focus is more directed to the patients than their colleagues or themselves. Over the period of data collection, a heat stress level of 4 or more for unacclimatised persons was classified for 46.9% of the 3229 readings. Under these conditions, periods of 15–45 min of break per 1 h of work, and enhanced water consumption are strongly encouraged.

**Fig. 3:**
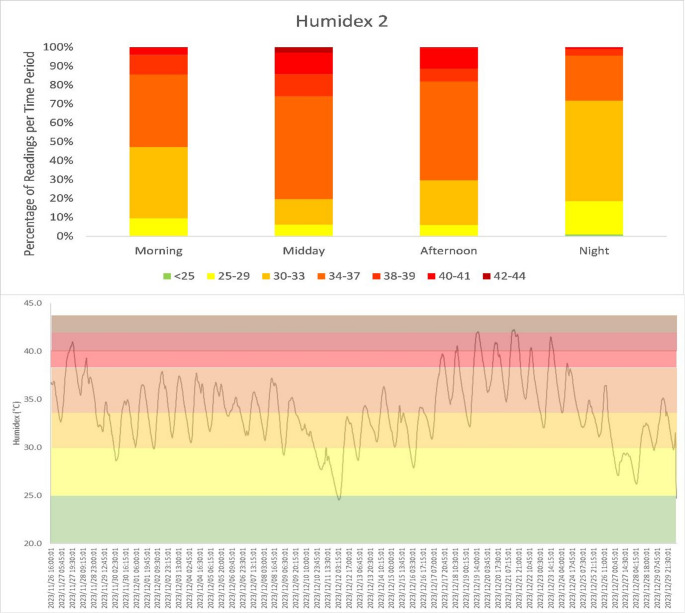
Incidence of each threshold level of heat stress risk as (A) a proportion of all readings and (B) time-series, using Humidex 2 classifications

Having demonstrated the prevalence of heat stress conditions in the early summer month in Klerksdorp, particularly for the unacclimatised person, it is of value to explore the times of day where heat stress is most common. This can facilitate shift allocation and rostering that aims to minimise prolonged exposure to heat stress and to optimise adequate quality sleep during cooler periods. Incidences of no heat risk for the period of data collection are restricted to the nighttime (Fig. 3). Conversely, cases of the most extreme (level 7) heat stress occur during midday. Level 6 heat stress, by contrast, is experienced across all four times of the day, with most frequent prevalence during midday. The greatest incidence of heat stress which requires intervention is experienced during midday and the afternoon.

**Fig. 4:**
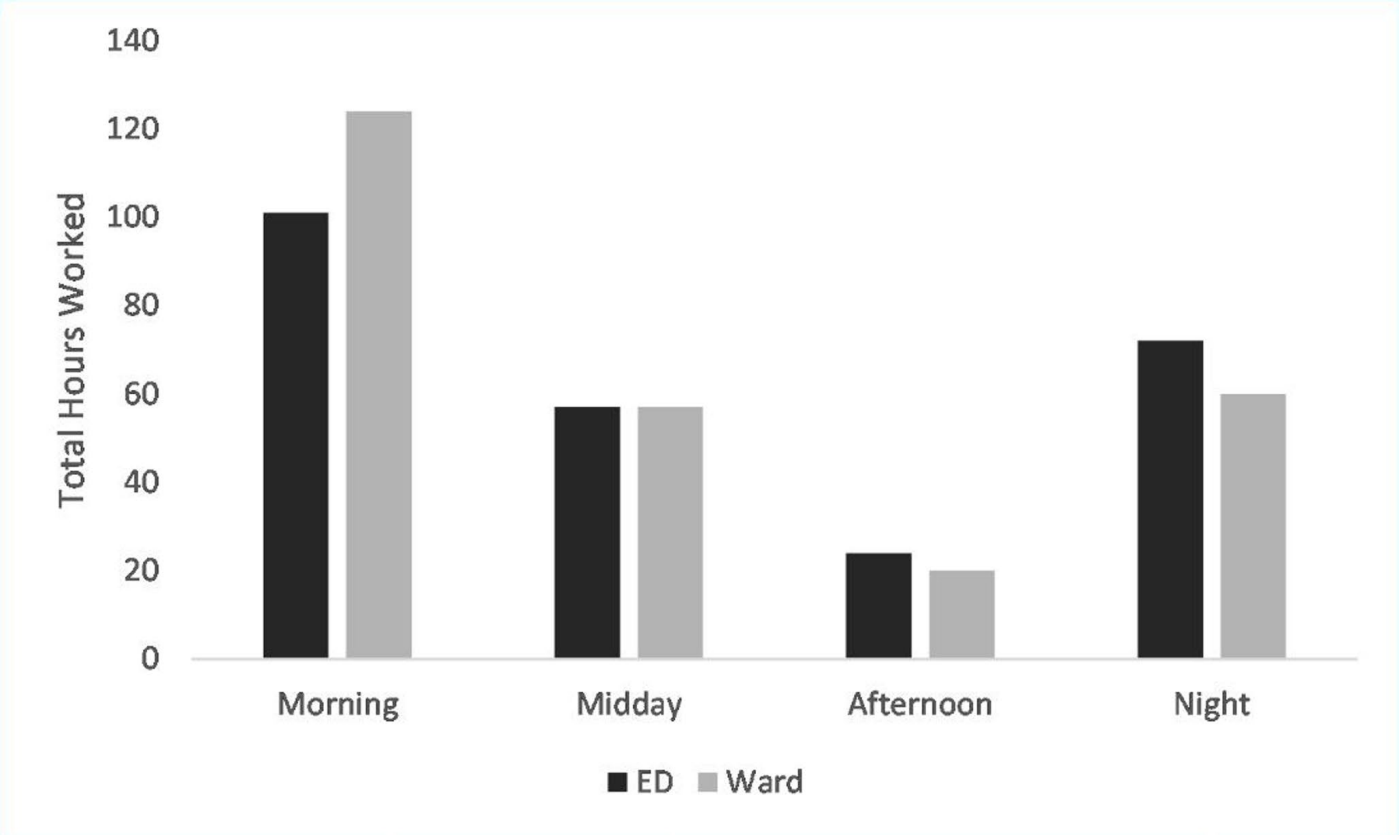
Total hours worked in a single calendar month from example rosters for the ED and the Ward based doctors

Regarding the timing of periods of physical exertion, sample rosters are valuable to explore the feasibility of shorter shifts during the times of day when doctors would be exposed to higher heat stress. Figure 4 shows that the majority of hours worked based on a sample roster from a calendar month during the study period are in the morning and the night time periods. This is true for the doctors working in the ED and those working in the wards, both of which include registrars rotating from Johannesburg for their respective training programmes. When comparing this to the periods of highest heat stress demonstrated in Fig. 3, it is evident that the doctors are working more hours in periods of lower heat stress risk. However, the greater concern in this study, given the large proportion of night hours worked, would be the potential for poor daytime sleep quality due to heat stress in the day following the night shifts.

To triangulate the results of the Humidex analysis, the risk of thermal stress is further quantified and classified using the Heat Index. These results indicate a 10.78% incidence of conditions considered to be ‘low risk’, and the majority (70.39%) classified as ‘caution’ (Table 3). Under the ‘caution’ classification, there is particular concern during periods of prolonged exposure. The classification of ‘extreme caution’ characterises 18.83% of the meteorological readings. None of the Heat Index scores from the measured data were classified as ‘danger’ or ‘extreme danger’. As a feature of the same meteorological variables, air temperature and relative humidity, the time characteristics are consistent across the two indices.

**Table 3:**
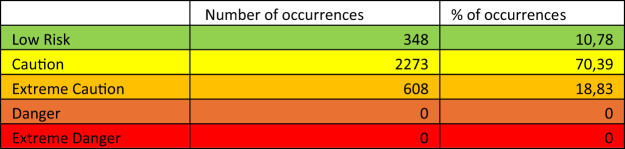
Frequency of occurrence of each category of Heat Index stress classification

## Discussion

### Significance of findings

The results of this study confirm that there is a discernible risk of heat stress in Klerksdorp in the early summer months of November and December, particularly to unacclimatised workers who are residing in temporary accommodation. While acclimatization is argued to be most difficult in the early summer (Lee et al. 2014), the risk of heat stress would persist, if not heighten, through the peak of the summer season in January and February (van der Walt and Fitchett 2020), and would be exacerbated during heat waves (van der Walt and Fitchett 2021; Mbokodo et al. 2023; Kapwata et al. 2024). This poses a particular threat to registrars who are rotating through Klerksdorp for a period of one to four months, particularly when the rotation takes place during the summer. Doctors living in temporary accommodation for short periods, often with duplicated accommodation costs, are unlikely to have the financial capital or the rental agreements to invest in air conditioners for their residence and would suffer the impact of sleep loss due to high night time temperatures (Minor et al. 2022). The impacts of heat stress on concentration levels, decision-making (De La Cruz et al. 2021), risk-taking (Syndicus et al. 2018) and aggression (Potgieter et al. 2022) are of concern in the fast-paced and stressful environment of the hospital. While this study uses in situ meteorological data, and computed thermal stress indices and classifications for a single residential equation, it confirms the risk of heat stress in that setting, and is likely representative of more widespread heat risk in Klerksdorp to unacclimatized workers.

The provision of healthcare in the hospital setting necessitates that staff are physically present at all times. The majority of junior doctors, including rotating registrars, working in the public sector in North West Province work 64–80 h of overtime hours per month as per the commuted overtime contract; the rostering of these overtime hours differs between departments (Fig. 4) but necessitates that night work is performed and therefore an amount of rest in the day will be taken. The combination of reduced sleep hours experienced by night shift workers as compared to day workers (Åkerstedt 2003), and reduced sleep hours experienced when exposed to higher ambient temperatures (Minor et al. 2022), results in sleep deprivation experienced in those doctors who are trying to rest after a night shift. The benefit of working more hours in the morning and the night periods would be the reduced exposure to heat stress during periods of exertion (Figs. 3 and 4). A strategy to minimise the dual effects of working in high heat stress environments and the reduced sleep duration and quality due to high ambient heat would be to consider a shift system that allows for greater periods of rest. In many countries limits have been placed on maximum shift duration due to concerns about the effect of sleep deprivation doctors’ health (Rodrigues et al. 2023). Similarly, in areas where there is a risk of heat stress, shifts could have limits placed on them or standardised rest periods worked into the roster, so as to minimise the effect on the doctors.

### Limitations and avenues for ongoing research

This study is based on data collected in situ in a residential environment, and reflects the challenges in acclimatization that can face doctors rotating specifically from Johannesburg to Klerksdorp in the early summer. The actual temperatures experienced by all individual healthcare workers at a microclimatic cale are not necessarily reflected by the temperature readings obtained, as there is no personal monitoring data recorded. The results indicating a discernible level of heat stress in early summer would suggest a high likelihood of more severe, prolonged and frequent heat stress during the mid-summer. These results are for a specific set of indoor conditions, although at a residential location frequented by rotating medical registrars, and of a building design and level of infrastructure common to the city. However, more widespread data logging in future would provide further insight into the role of air-conditioning, where available. Future research exploring year-round in situ meteorological conditions would be valuable in quantifying this, especially in environments such as Doctors Quarters (DQ), where consecutive groups of rotating doctors reside. Repeating this study for other rural rotation locations across South Africa would similarly be valuable in contributing to policy adaptation. Additionally, research exploring doctors’ experiences of heat stress, and their mitigation strategies, would be valuable in understanding the extent of the risk of heat stress. While not the focus of this study, continued research on heat within medical facilities (cf. Wright et al. 2017) would allow for ongoing policy intervention to ensure the thermal wellbeing of medical practitioners while at work, and of the patients they treat.

## Future directions and conclusion

While the focus of this study is the effect of heat stress on unacclimatised registrars in the domestic setting, it does highlight the need for further work in analysing the risks posed in the work place as well, as this would be a time of greater physical exertion. The risks of heat stress during work and at home can be mitigated through both physical and social adaptation (Ebi and Semenza 2008). The most effective means of infrastructural adaptation is through the installation and upgrading of efficient Heating, Ventilation and Air Conditioning (HVAC) systems; the design of which has received research focus in hospital settings in recent years (Lenzer et al. 2020; Del Regno et al. 2023). Here the intervention would benefit both the medical staff and the patients while at the hospital, and the potential for adequate sleep of staff at home (Lenzer et al. 2020). However, such HVAC systems are often costly, and energy intensive, which is challenging in the South African context particularly during loadshedding (scheduled power interruptions), when hospitals rely on generators for their electricity supply, and by necessity do not prioritise any means of temperature control during those periods (Laher et al. 2019). Furthermore, the effect of utilisation of HVAC systems on worsening climate change through greenhouse gas emissions has resulted in the National Department of Health recommending against their use in the Heat Health Action Guidelines (NDoH 2020). Social adaptation refers to the behavioural changes that can be made to minimise the impacts of climate on people (Burton et al. 2009). At a municipal scale, larger cities, such as Johannesburg, have started to develop heat health plans for heat wave events and longer-term climate change (Nana et al. 2019); no such plan has yet been developed for Klerksdorp. However, at the scale of individual hospitals, adaptation plans can be developed for the summer months, particularly in terms of the scheduling of shifts to minimise the duration of time worked through the hottest periods of the day. There is an extensive literature on statistical approaches to optimise ED shift scheduling to minimise patient waiting time (El-Rifai et al. 2015), maximise training in academic hospitals (Schuh et al. 2021), improve practitioner wellbeing (Smith-Coggins et al. 1997), and achieve combinations thereof (Chawasemerwa et al. 2018). The results of this study, indicating that the highest periods of heat stress risk are in the afternoon, and the specific Humidex output scores, would form a valuable input into such models. At an even finer resolution, individual social adaptation can be implemented through using the Canadian Centre for Occupational Health and Safety classifications of response for each level of heat stress, with particular relation to cool water intake and rest periods (CCOHS 2024). Long-term adaptation may also involve the introduction of acclimatisation periods for rotating doctors, and constraining rotations to hotter cities to autumn, winter and spring. To conclude, we emphasise the importance of recognising the heat stress posed to medical practitioners working in emergency departments during the hotter months, and advocate for more proactive adaptation strategies.

## Data Availability

Data are available on request.

## Code availability

n/a.
